# Depressive symptoms can amplify embarrassment in essential tremor

**DOI:** 10.1186/s40734-016-0039-6

**Published:** 2016-07-15

**Authors:** Elan D. Louis, Stephanie Cosentino, Edward D. Huey

**Affiliations:** Division of Movement Disorders, Department of Neurology, Yale School of Medicine, Yale University, LCI 710, 15 York Street, PO Box 208018, New Haven, CT 06520-8018 USA; Department of Chronic Disease Epidemiology, Yale School of Public Health, Yale University, New Haven, CT USA; Center for Neuroepidemiology and Clinical Neurological Research, Yale School of Medicine, Yale University, New Haven, CT USA; Department of Neurology, College of Physicians and Surgeons, Columbia University, New York, NY USA; Division of Geriatric Psychiatry, Department of Psychiatry, College of Physicians and Surgeons, Columbia University, New York, NY USA; G.H. Sergievsky Center, College of Physicians and Surgeons, Columbia University, New York, NY USA; Taub Institute for Research on Alzheimer’s Disease and the Aging Brain, Columbia University, New York, NY USA

**Keywords:** Essential tremor, Non-motor, Depression, Embarrassment, Clinical, Treatment

## Abstract

**Background:**

Embarrassment can be a considerable problem for patients with essential tremor (ET) and is a major motivator for treatment. Depression is also a common feature of ET; as many as 35 % of patients report moderate to severe depressive symptoms. Our goal was to assess the associations between these motor and psychosocial factors (tremor, depression, embarrassment) in ET, with a particular interest in more fully assessing the possible association between depression and embarrassment.

**Methods:**

Ninety one ET cases (age 70.4 ± 12.8 years) enrolled in a prospective, clinical-epidemiological study. Depressive symptoms were assessed with the Center for Epidemiological Studies Depression Scale (CESD-10, 0–30 [maximum]), embarrassment, with the Essential Tremor Embarrassment Assessment (ETEA, 0–70 [maximum]), and action tremor, with a detailed in-person neurological examination.

**Results:**

Higher CESD-10 score was significantly associated with higher ETEA score (*p* = 0.005), but not with increasing tremor severity (*p* = 0.94). In stratified analyses, cases with no or minimal depressive symptoms had the lowest ETEA scores, cases with moderate depressive symptoms had intermediate ETEA scores, and cases with severe depressive symptoms had the highest ETEA scores (*p* = 0.01). Furthermore, at each level of tremor severity, cases with more depressive symptoms had more embarrassment.

**Conclusions:**

Depressive symptoms seem to be more than a secondary response to the tremor in ET; they seem to amplify the level of embarrassment and, in addition to their own importance, seem to be a driver of other important clinical outcomes. Earlier treatment of depressive symptoms in ET patients could lessen the burden of secondary embarrassment.

## Background

According to some estimates, 60 % of essential tremor (ET) patients report embarrassment surrounding their tremor [1]. Embarrassment, classified as a “self-conscious” emotion along with shame and guilt, and in contrast to “basic” emotions such as anger and joy [2], often arises when someone violates a social rule or expectation [3]. Embarrassment is associated with autonomic reactivity including increased heart rate, blood pressure, and sweating and behavioral reactions including gaze avoidance, regret signaling, and avoidance of others [3]. Embarrassment about tremor is a considerable problem for patients with ET [1, 4–7]. It is one of the two main motivators for ET patients to initiate medical therapy [1, 4–6] and it is a particularly strong predictor of receptivity to deep brain stimulation (DBS) surgery among patients with ET [8]. Moreover, feelings of embarrassment can lead to avoidance of social situations and social isolation [9]. Indeed, handling embarrassment and the social effects of tremor has been highlighted by ET patients as one of the top issues not being addressed in their care [10].

Depressive symptoms and depression have been associated with ET in numerous case–control studies [11–17]; according to some estimates, as many as 35 % of patients report moderate to severe depressive symptoms [17]. Similar to embarrassment, depression in ET is often viewed as a secondary response to the disabling condition [11, 14]. Overall; however, depression has not been well studied in ET [11], and there is emerging evidence that depression may be a primary feature of the disease, preceding motor symptoms [18]. This suggests that depression itself could drive other clinical outcomes in ET, such as embarrassment, rather than representing a passive response to the motor symptoms.

Although tremor, embarrassment, and depression may all occur in ET, and likely impact one another, the associations among them have not been the subject of previous analyses. The goal of these analyses was to assess the associations between these motor and psychosocial factors (ie, tremor, embarrassment, and depression) in individuals with ET, with a particular interest in more fully assessing the possible association between depression and embarrassment.

## Methods

### Participants and evaluation

As described previously, ET cases were enrolled in a clinical-epidemiological study of the epidemiology of movement disorders at Columbia University Medical Center (CUMC) [19, 20]. The large majority of cases were derived from two sources: (1) a computerized billing database of ET patients at the Neurological Institute of New York, CUMC, and (2) advertisements to members of the International Essential Tremor Foundation. One-hundred-forty-one cases were enrolled (2009–2014). During that time period, a formal assessment of embarrassment was added. Ninety-one cases were enrolled after the embarrassment assessment was added. Cases had all received a diagnosis of ET from their treating neurologist and were confined to a geographical area within 2 h driving distance of CUMC. Prior to enrollment, one of the authors (E.D.L.) reviewed the office records of identified patients; those with diagnoses of or physical signs consistent with other movement disorders were excluded.

The CUMC Internal Review Board approved of all study procedures. Written informed consent was obtained upon enrollment. Analysis of data was also approved by the Internal Review Board at Yale School of Medicine.

During the in-person evaluation, the trained research worker administered a series of structured clinical questionnaires (demographics, clinical features, medications, family history). The research worker also administered the Center for Epidemiological Studies Depression Scale (CESD-10) (0–30 [higher scores indicate greater depressive symptoms]) [21]. The CESD is a self-report measure of 10 questions about the frequency of experiencing (0 to 3 for each item) different depressive symptoms. It is a reliable and valid instrument [22]. In addition, the Essential Tremor Embarrassment Assessment (ETEA), an assessment of tremor-related embarrassment (range = 0–70 [maximal embarrassment]) [15] was administered to ET patients [4]. The ETEA, which is a valid and reliable instrument [4], comprises 14 questions that assess overall embarrassment and its effects on the patient’s desire for tremor medication, as well as embarrassment in a variety of situations (eg, eating in public, speaking in front of a group, social situations). Each item is rated from 0 to 5, with higher scores indicating greater embarrassment [4].

All cases underwent a standardized videotaped tremor examination, which included tests of postural and kinetic tremors and assessments for the presence of other involuntary movements. The aim was to use the videotape to carefully validate ET diagnoses using rigorous research-grade diagnostic criteria [23]. Thus, each videotape was reviewed by a senior neurologist specializing in movement disorders (E.D.L.) who confirmed the ET diagnoses using Washington Heights-Inwood Genetic Study of ET (WHIGET) diagnostic criteria (moderate or greater amplitude kinetic tremor [tremor rating ≥2] during three or more tests or a head tremor, in the absence of Parkinson’s disease, dystonia or another cause) [23]. The neurologist also rated postural and kinetic tremor (range = 0–3) during 12 videotaped tests and computed a total tremor score (range = 0–36).

### Statistical analyses

Data were analyzed in SPSS (Version 22.0). Chi-square tests were used to assess associations within categorical data. Total tremor score, ETEA score and CESD-10 score were all normally distributed (Kolmogorov-Smirnov Test *p* values = 0.67, 0.70, 0.35, respectively); hence, parametric tests (eg, Pearson’s r) were used when assessing these variables.

Several CESD-10 cut-offs have been recommended for depression, including a score ≥10 [21], and a more conservative score ≥20 [24]. To incorporate both sets of recommendations, as in a prior set of analyses [25], we divided cases into three groups based on their CESD-10 score: 0–9 (no or minimal depressive symptoms), 10–19 (moderate depressive symptoms), ≥20 (severe depressive symptoms). Scores > 20 have high sensitivity and specificity for the diagnosis of Major Depressive Disorder as defined in the DSM [26–28]. To derive strictly mathematical cut-points, CESD-10 scores were also stratified into quartiles (≤3, 4–7, 8–12, ≥13).

Linear regression models were used to assess the associations between variables.

## Results

The 91 ET cases had a mean age of 70.4 ± 12.8 years and a mean tremor duration of 36.9 ± 18.7 years. The mean CESD-10 score was 9.5 ± 6.2 (range = 0–26) (Table 1), with 47 (51.6 %) having no or minimal depressive symptoms, 37 (40.7 %) having moderate depressive symptoms and 7 (7.7 %) having severe depressive symptoms. Six (6.6 %) cases had CESD-10 scores > 20, of whom one (1.1 %) was also taking an antidepressant medication. The mean ETEA score was 24.2 ± 16.9 (range = 0–61).

**Table 1:** Demographic and clinical characteristics of 91 ET cases

Age in years	70.4 ± 12.8 (range = 33–96)
Female gender	47 (51.6)
Education in years	16.2 ± 2.8
Non-Hispanic white race	86 (94.5)
Age of onset of tremor in years	37.7 ± 18.1
Tremor duration in years	36.9 ± 18.7
Family history of:	
ET	28 (30.8)
ET or tremor	58 (63.7)
Total tremor score	20.6 ± 5.9
Head (neck) tremor on examination	32 (35.2)
Voice tremor on examination	24 (26.4)
CESD-10 score	9.5 ± 6.2 (range = 0–26)
CESD-10 score	
0–9 (no or minimal depressive symptom category)	4.7 ± 2.7 (n = 47)
10–19 (moderate depressive symptom category)	13.0 ± 2.5 (*n* = 37)
≥ 20 (severe depressive symptom category)	23.1 ± 2.3 (*n* = 7)
CESD-10 score	
≤ 3 (lowest quartile)	1.4 ± 1.2
4–7 (second quartile)	5.4 ± 1.2
8–12 (third quartile)	10.2 ± 1.2
≥ 13 (highest quartile)	17.1 ± 4.1
ETEA score	24.2 ± 16.9 (range = 0–61)

Values represent mean ± standard deviation or number (percentage)

CESD-10 (Center for Epidemiological Studies Depression Scale), ETEA (Essential Tremor Embarrassment Assessment)

**Fig. 1:**
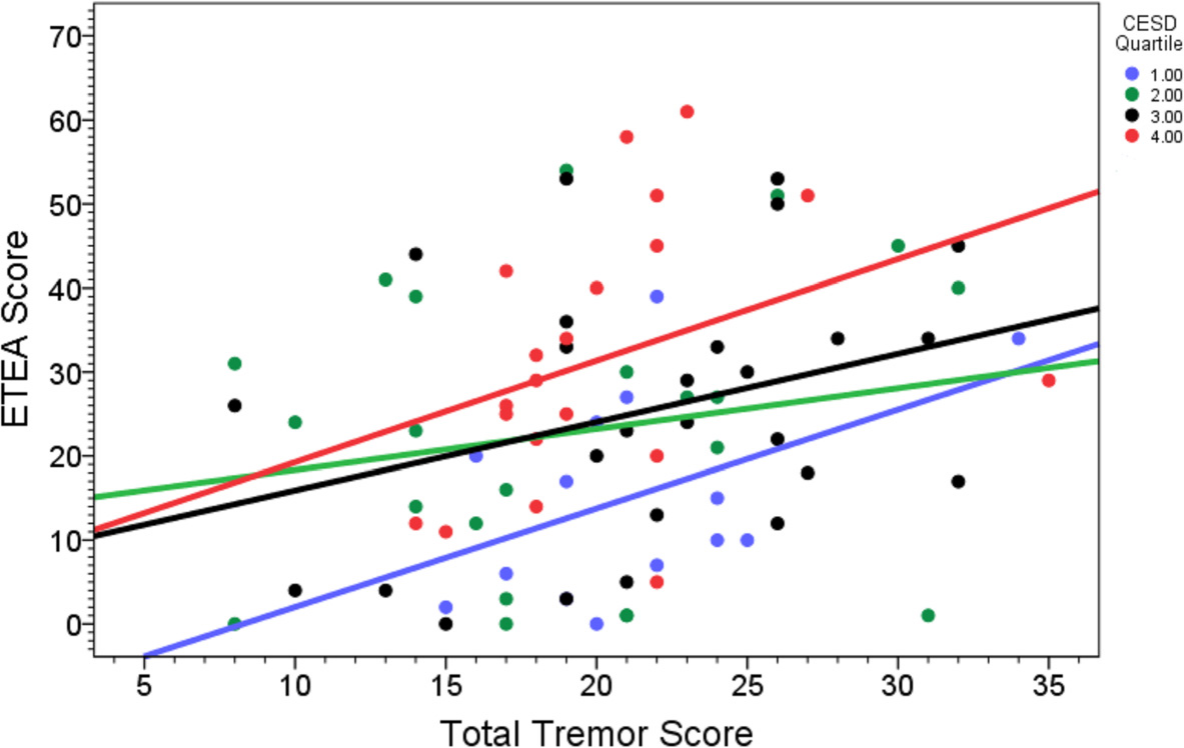
ETEA score by total tremor score in each CESD-10 quartile. At each level of tremor severity (ie, at each total tremor score), higher level of depressive symptoms was associated with more embarrassment; thus, cases in the lowest CESD-10 quartile (ie, fewest depressive symptoms) had the lowest levels of embarrassment and cases in the highest CESD-10 quartile (ie, most depressive symptoms) had the highest levels of embarrassment

Higher total tremor score was associated with higher ETEA score (Pearson’s *r* = 0.27, *p* = 0.016 and see Fig. 1); however, higher total tremor score was not associated with higher CESD-10 score (Pearson’s *r* = 0.008, *p* = 0.94).

**Fig. 2:**
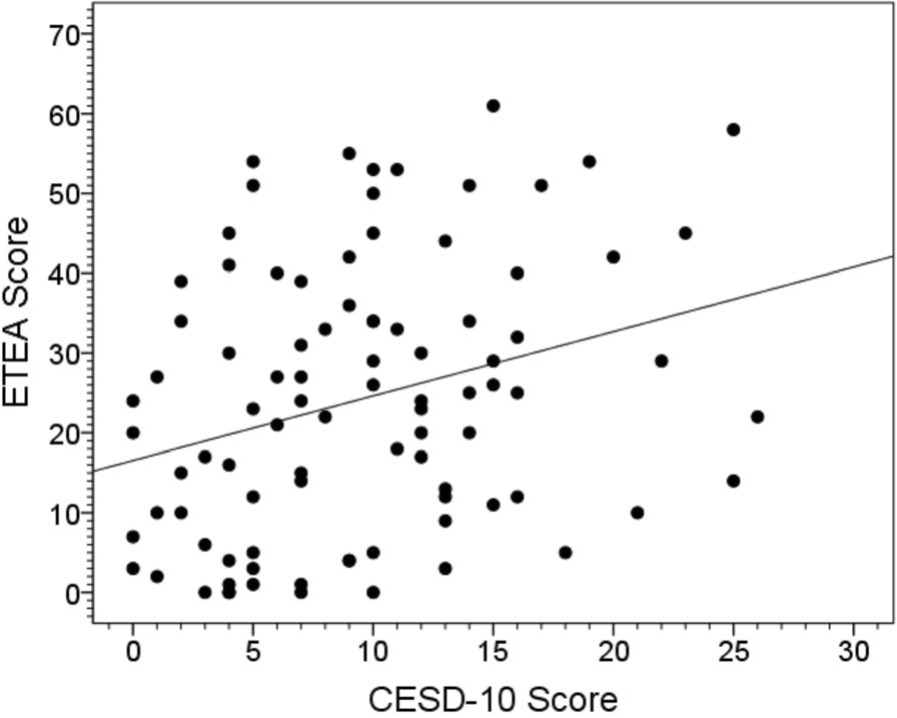
ETEA score by CESD-10 score. Higher CESD-10 score was associated with higher ETEA score (Pearson’s *r* = 0.29, p = 0.005)

Higher CESD-10 score was associated with higher ETEA score (Pearson’s *r* = 0.29, *p* = 0.005, Fig. 2). Furthermore, cases with no or minimal depressive symptoms had the lowest ETEA scores, cases with moderate depressive symptoms had intermediate ETEA scores, and cases with severe depressive symptoms had the highest ETEA scores (*p* = 0.01, Table 2). Similarly, there was a significant association between CESD-10 score quartile and ETEA score (*p* = 0.001, Table 2).

**Table 2:** Association between CESD-10 and ETEA scores in 91 ET cases

	ETEA score
CESD-10 score category	
0–9 (no or minimal depressive symptoms)	19.9 ± 16.4
10–19 (moderate depressive symptoms)	28.4 ± 16.4
≥ 20 (severe depressive symptoms)	31.4 ± 17.6
	*p* = 0.01^a^
CESD-10 score quartiles	
≤ 3 (lowest quartile)	15.3 ± 12.2
4–7 (second quartile)	20.2 ± 17.3
8–12 (third quartile)	26.6 ± 16.5
≥ 13 (highest quartile)	31.6 ± 16.7
	*p* = 0.001^b^

Values represent mean ± standard deviation or number (percentage)

CESD-10 (Center for Epidemiological Studies Depression Scale), ETEA (Essential Tremor Embarrassment Assessment)

^a^Linear regression analysis with ETEA score as dependent variable and CESD score category as the independent variable

^b^Linear regression analysis with ETEA score as dependent variable and CESD score quartile as the independent variable

At each level of tremor severity (ie, at each total tremor score), higher level of depressive symptoms was associated with more embarrassment; thus, cases in the lowest CESD-10 quartile (ie, fewest depressive symptoms) had the lowest levels of embarrassment and cases in the highest CESD-10 quartile (ie, most depressive symptoms) had the highest levels of embarrassment (Fig. 1).

Cases with severe depressive symptoms (CESD-10 score ≥20) had higher ETEA scores than those with fewer depressive symptoms, despite the fact that they had similar levels of tremor severity (Table 3). Indeed, mean level of embarrassment was 50 % higher in cases with severe depressive symptoms than those with no or minimal depressive symptoms (31.4 vs. 19.9, Table 3) despite nearly identical total tremor scores (19.0 vs. 19.6, Table 3). This was associated with greater medication usage; 7 of 7 (100 %) cases with severe depressive symptoms had taken medication for tremor vs. 28/47 (59.6 %) of those with no or minimal depressive symptoms (chi-square test = 4.37, *p* = 0.037, Table 3).

**Table 3:** CESD-10 score category, total tremor score, ETEA score and medication usage in 91 ET cases

CESD-10 score category	n	Total tremor score	ETEA score	Number (%) who had been prescribed medication for tremor
0–9 (no or minimal depressive symptoms)	47	19.6 ± 6.2	19.9 ± 16.4	28 (59.6)
10–19 (moderate depressive symptoms)	37	22.1 ± 5.8	28.4 ± 16.4	24 (64.9)
≥20 (severe depressive symptoms)	7	19.0 ± 2.0	31.4 ± 17.6	7 (100)
		*p* = 0.36^a^	*p* = 0.01^b^	

Values represent mean ± standard deviation or number (percentage)

CESD-10 (Center for Epidemiological Studies Depression Scale), ETEA (Essential Tremor Embarrassment Assessment)

^a^Linear regression analysis with total tremor score as dependent variable and CESD score category as the independent variable

^b^Linear regression analysis with ETEA score as dependent variable and CESD score category as the independent variable

In a subgroup analysis of 58 patients with family history of ET or tremor, higher CESD-10 score was associated with higher ETEA score (Pearson’s *r* = 0.30, *p* = 0.02). Furthermore, cases with no or minimal depressive symptoms had the lowest ETEA scores (*n* = 28, 21.9 ± 17.6), cases with moderate depressive symptoms had intermediate ETEA scores (*n* = 25, 28.6 ± 16.4), and cases with severe depressive symptoms had the highest ETEA scores (*n* = 5, 36.8 ± 18.2) (linear regression analysis, *p* = 0.04).

## Discussion

Tremor, depression, and embarrassment may co-occur in many ET patients, making this an important constellation of motor and psychosocial factors. Hence, it is surprising that the associations between these factors have not been delineated previously. In the current study, higher depressive symptom scores were associated with significantly greater levels of embarrassment (*p* = 0.005). Indeed, cases with no or minimal depressive symptoms had the lowest embarrassment scores, cases with moderate depressive symptoms had intermediate embarrassment scores, and cases with severe depressive symptoms had the highest embarrassment scores (*p* = 0.01). Cases with severe depressive symptoms (CESD-10 score ≥20) had higher ETEA scores than those with fewer depressive symptoms, despite the fact that they had similar levels of tremor severity. Indeed, level of embarrassment was 50 % higher in cases with severe depressive symptoms than those with minimal depressive symptoms despite nearly identical total tremor scores.

As noted above, at each level of tremor severity, cases who had more depressive symptoms had more embarrassment. While it is conceivable that greater embarrassment could lead to more depression, it is more plausible that the converse is the case, that is, that depressive symptoms are amplifying the level of embarrassment. The CESD-10 scale measures the full range of depressive symptoms including somatic symptoms such as poor appetite, decreased energy, psychomotor retardation, and insomnia. Embarrassment is unlikely to be the direct cause of any of these depressive symptoms. Depression often acts as an amplifier of related symptoms in psychiatric disorders. Depression is associated with an increase in the distress and disability associated with chronic pain [29]. Self-conscious emotions including shame, guilt, and embarrassment increase, often dramatically, during an episode of major depressive disorder [30].

More broadly, these data also suggest that depressive symptoms are a driver of other important clinical outcomes in ET rather than merely a passive, secondary response to tremor. Depressive symptoms and/or depression have been reported as more prevalent in ET cases than controls in numerous studies [11–17]. Indeed, in the current study, more than half of the cases had moderate or severe depressive symptoms. Despite its high prevalence, the causes, effects and natural history of depression in ET have not been studied in any detail [11], although in a prior set of analyses, we showed that depressive symptoms were a strong predictor of tremor-related quality of life in ET [25]. Furthermore, its relationship to the motor features of ET may be complex. Emerging evidence suggests that depression may even be a primary feature of ET, preceding the motor features [18]. This finding has precedent in other movement disorders, including Huntington’s disease and Parkinson’s disease [31, 32]. Studies that examine the clinical correlates of depression and its relationships with other disease features in ET are therefore of importance.

One clinical implication of the current findings is that they increase the importance of treating depressive symptoms in patients with ET as, in addition to reducing depressive symptoms, it may reduce embarrassment as well. Embarrassment is a considerable problem for ET patients [1, 4–6]. It is one of the two main motivators for initiating medical therapy [1, 4–6] and can further lead to avoidance of social situations and social isolation [9]. Indeed, handling the embarrassing social effects of tremor has been highlighted by patients as one of the top issues not being addressed in their current care [10]. While the effect of depression treatment has not been specifically studied in ET patients, the effect size for the treatment of depressive symptoms with antidepressant medication in patients with Parkinson’s disease is moderate [33]. The findings of the current study also suggest that the treatment of depression with psychotherapy in patients with ET should assess and target embarrassment as well as the more usual depressive symptoms. In ET patients with depression and prominent embarrassment, referral to a psychotherapist who utilizes a symptom-based therapy method such as Cognitive-Behavioral Therapy (CBT) or Problem-Solving Therapy (PST) could be preferable to insight-oriented psychodynamic therapy.

Our cases had a mean CESD-10 score of 9.5 ± 6.2. A feature of this clinical-epidemiological study, but not of these analyes, was the enrollment of control subjects of similar age (*n* = 177, 74.9 ± 9.5 years). The mean CESD-10 score of these controls was only 6.7 ± 4.6, a value that was lower than that of our cases (*t* test = 3.84, *p* <0.001), indicating a higher burden of depressive symptoms among our cases, as has been reported in ET in the past [11–17].

This study should be interpreted within the context of certain limitations. First, the study was cross-sectional, so that we are not able to directly address issues of causality. Second, the sample size was modest and this may have limited our ability to detect associations. However, the study was able to detect significant main effects between several of the key variables. Third, depressive symptoms were assessed with a brief, validated screening instrument; it is possible that more in-depth psychiatric assessments could have uncovered additional associations of interest. For example, the relationship between symptoms of anxiety, including social anxiety, and embarrassment and the relationship between ET and other self-conscious emotions. In particular, anxiety is also a common feature of patients with ET [9, 17] and it could impact upon levels of embarrassment. Future studies could further assess such relationships. Fourth, we did not collect data on past history of depression in our cases; such information could have supplemented the data we collected on current depressive symptoms.

The study also had several strengths. First, to our knowledge, it is the only study to have assessed this particular gap in knowledge and these particular associations. Second, this was not a retrospective study or chart review; the study cohort was enrolled prospectively, with a standardized assessment.

## Conclusions

In summary, we found that cases with severe depressive symptoms had higher embarrassment scores than those with fewer depressive symptoms, despite the fact that they had similar levels of tremor severity. Furthermore, greater tremor severity was not associated with more depressive symptoms. One interpretation of these data is that depressive symptoms are more than a secondary response to the tremor in ET; in addition to being clinically important in themselves, they also seem to drive other important outcomes such as embarrassment. Earlier treatment of depressive symptoms in ET patients might be a strategy to lessen the burden of secondary embarrassment.

## Abbreviations

(CES-D), Center for Epidemiological Studies Depression Scale; (CBT), Center for Epidemiological Studies Depression Scale; (CUMC), Columbia University Medical Center; (DBS),Deep brain stimulation; (ET), Essential tremor; (ETEA), Essential Tremor Embarrassment Assessment; (PST), Essential Tremor Embarrassment Assessment; (WHIGET), Washington Heights-Inwood Genetic Study of ET
